# Adherence to oral anticoagulation measured by electronic monitoring in a Belgian atrial fibrillation population

**DOI:** 10.1007/s00392-023-02261-w

**Published:** 2023-07-27

**Authors:** Lieselotte Knaepen, Michiel Delesie, Johan Vijgen, Paul Dendale, Joris Ector, Lien Desteghe, Hein Heidbuchel

**Affiliations:** 1 Antwerp University Hospital, Drie Eikenstraat 655, 2650 Edegem, Belgium; 2 Research Group Cardiovascular Diseases, University of Antwerp, Prinsstraat 13, 2000 Antwerp, Belgium; 3 Faculty of Medicine and Life Sciences/LCRC, Hasselt University, Martelarenlaan 42, 3500 Hasselt, Belgium; 4 Heart Center Hasselt, Jessa Hospital, Stadsomvaart 11, 3500 Hasselt, Belgium; 5 Department of Cardiology, University Hospitals Leuven, Leuven, Belgium

**Keywords:** Atrial fibrillation, Oral anticoagulation, Therapy adherence, Telemonitoring

## Abstract

**Introduction:**

Stroke prevention using oral anticoagulation (OAC) is the first management priority in atrial fibrillation (AF). Despite the importance of good therapy adherence, real-world adherence is still suboptimal. Patient education and adherence monitoring with new technologies are recommended. The main purpose of this sub-analysis of the AF-EduCare trial was to evaluate the effect of personalized follow-up strategies on adherence to OAC.

**Methods:**

Regimen adherence was monitored by the electronic Medication Event Monitoring System cap at the start of the trial (M1) and after 12 months (M2), each for three months. Patients were part of one of three education groups (In-person, Online or App-based) or the standard care (SC) group. All are qualified for OAC therapy.

**Results:**

A total of 768 patients were evaluated (11.8% SC vs. 86.8% any education group, mean age: 70.1 ± 7.9 years). Patients were taking non-vitamin K OAC (once daily 53.8%; twice daily 35.9%) or vitamin K antagonists (9.4%), equally distributed over the different study arms (*p* = 0.457). Mean therapy adherence was high (M1:93.8 ± 10.8%; M2:94.1 ± 10.1%). During both monitoring periods, the education group scored significantly higher than SC (M1:94.2 ± 10.0% vs. 91.3 ± 15.0%; *p* = 0.027; M2:94.4 ± 9.3% vs. 91.6 ± 14.0%; *p* = 0.006). More patients in the In-person and Online groups were able to keep or improve their adherence to > 90% compared to the SC.

**Conclusion:**

Overall adherence to OAC in all study groups, even in SC, was very high, without attrition over time. Nevertheless, targeted education led to a small but significantly improved adherence compared to SC.

**Graphical Abstract:**

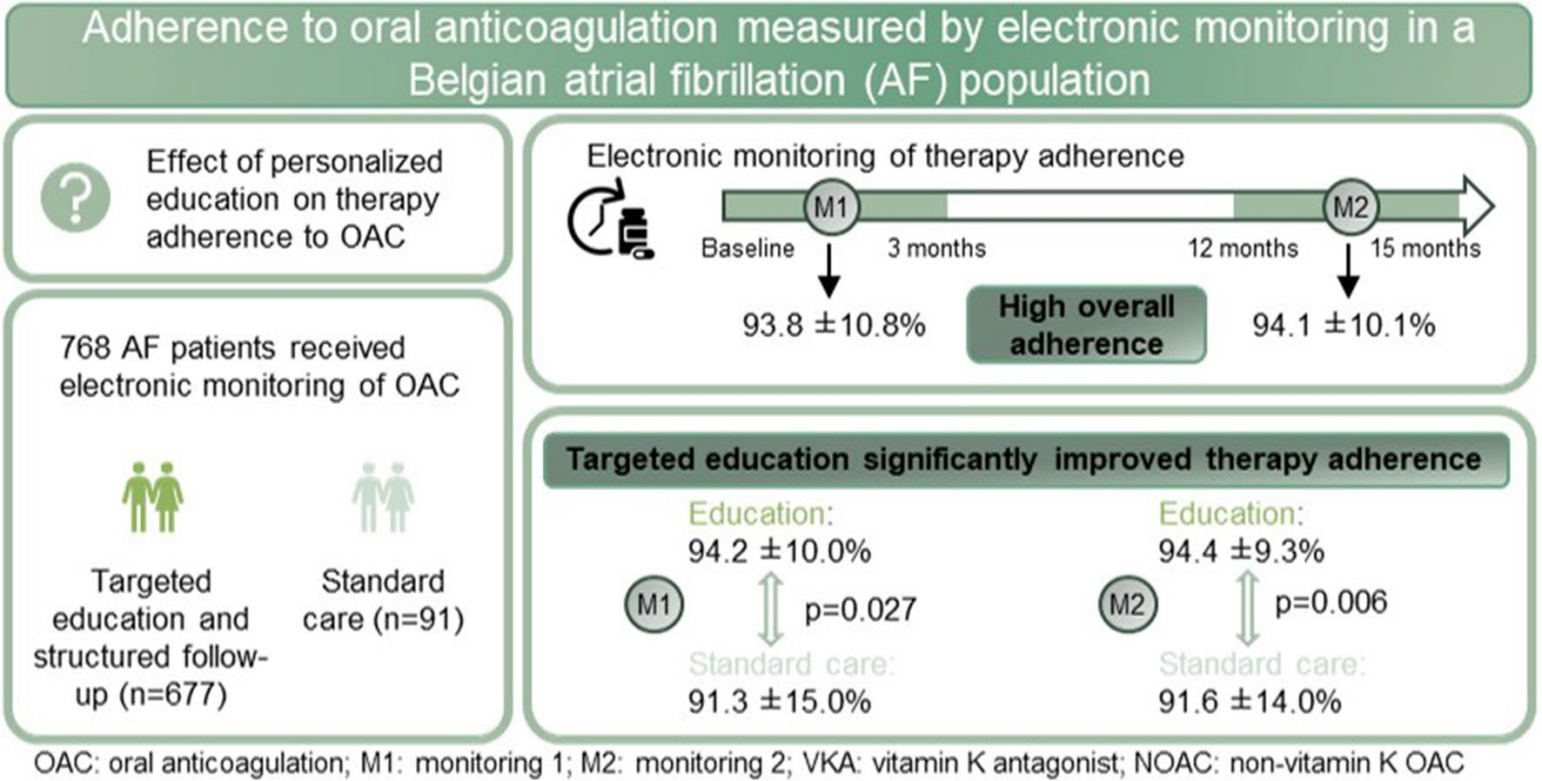

**Supplementary Information:**

The online version contains supplementary material available at 10.1007/s00392-023-02261-w.

## Introduction

Treatment with oral anticoagulation (OAC) is one of the main pillars in managing atrial fibrillation (AF), as the risk of stroke is five times higher in patients with AF compared to persons without AF [1]. About 90% of AF patients take OAC, either vitamin K antagonists (VKA) or non-vitamin K antagonist oral anticoagulants (NOAC), the latter being now the preferred strategy (in the absence of mechanical prosthetic heart valves or moderate to severe mitral stenosis) [2, 3] Four NOACs are commercially available: the twice-daily taken (BID) apixaban or dabigatran, and the once-daily taken (OD) rivaroxaban or edoxaban. For optimal efficacy of these NOACs because of their short anticoagulation effect, strict adherence to the recommended dose schedule is essential to reduce the risk for thrombo-embolic and bleeding events in comparison with the VKAs [4].

However, real-world adherence to NOAC therapy is not always optimal. Recent reviews and meta-analyses of *Salmasi *et al*.* and *Ozaki *et al*.* showed that about 30% of AF patients are non-adherent and mean adherence ranges between 57 and 82% [5, 6]. It is clear that despite the convenience for patients of NOACs which do not require laboratory testing but require strict adherence, efforts are needed to monitor adherence and prevent non-adherence.

Besides patient education, electronic monitoring of medication intake can be used as a support tool to monitor medication intake, as suggested by the European Society of Cardiology (ESC) AF Management Guidelines and the European Heart Rhythm Association (EHRA) 2021 Practical Guide on the use of NOACs in AF patients [3, 7, 8]. Such measurements allow self-regulation of the intake habits of patients, which can lead to actions to optimize adherence.

Our aim was to investigate the effect of different educational interventions on regimen adherence to OAC (both VKA and NOAC) in a contemporary Belgian AF population.

## Methods

This study is part of a prospective, multicenter, randomized controlled trial executed in three Belgian hospitals (AF-EduCare study; NCT03707873 and AF-EduApp sub-study; NCT03788044) [9]. Within these trials the AF-EduCare approach was used, which is based on four aspects (discussed by Delesie et al.): (I) targeted education about AF and its treatment, (II) assessment of AF-related riks factors (RF) and informing patients on how to tackle these, (III) ensuring high therapy adherence with the use of electronic monitoring, and (IV) offering an easy way for patients to ask questions about AF and its management. The effect of different personalized AF education strategies (i.e., In-person, Online, or App-based education) on cardiovascular outcome of all types of AF patients compared to standard care (SC) is the primary outcome of the AF-EduCare trial. The AF-EduApp study was implemented as a sub-study in the AF-EduCare trial (Supplementary Fig. 1) with the AF-EduCare approach incorporated in the AF-EduApp application. Therapy adherence to OAC is the primary outcome of the AF-EduApp sub-study. With both trials having therapy adherence as outcome parameter (Supplementary Fig. 1), this paper focusses on the third pillar of the AF-EduCare approach (i.e., improving therapy adherence).

### Study population and design

The whole study protocol is already published [9]. However, inclusion of patients and the different study groups will be explained briefly. All types of AF patients (i.e., > 18 years, able to speak and read Dutch, not cognitive impaired and with a life expectancy > 1 year), hospitalized at the cardiology ward or coming for an outpatient visit could be included in the AF-EduCare/AF-EduApp study. After patients agreed to participate, the patients were randomly assigned to one of three intervention groups (In-person, Online, App-driven education), or SC. Patients in the In-person group were seen in the hospital, where a research nurse gave them targeted education. Patients in the Online and App groups, however, had access to either an online education platform (i.e., with AF information and possibility to fill-out questionnaires) or the in-house developed AF-EduApp application (containing 6 main modules [10], explained in Supplementary Annex 1), respectively.

### Intervention and data collection

One of the study interventions focuses on improving OAC therapy using an electronic Medication Event Monitoring System (MEMS, AARDEX Group, Liège, Belgium). Ambulatory or hospitalized AF patients randomized to the intervention groups, treated with a NOAC or VKA at baseline or initiated during the study, were monitored with the MEMS cap at the start of the study (Monitoring 1, M1) and again after one year (Monitoring 2, M2), each time for a period of three months. The app-based group was monitored during the whole 15 months; read-outs at the same time points were used as in the other groups. An LCD screen on the MEMS cap displays the number of openings of the medication bottle over 24 h, providing direct patient feedback about daily intake. Every bottle opening is stored as data in the MEMS cap build-in memory card, which was read out by the study team after the three-month monitoring period at the 3- and 15-months follow-up visits. Since VKA doses are variable and since dabigatran has to be stored in the original package to protect it from moisture, a proxy medication was chosen to measure adherence for these drugs, i.e., another oral drug of importance that the patient had to take OD or BID at the same time(s) of the day as VKA or dabigatran.

Regimen adherence was calculated as the number of days on which one bottle opening (in case of rivaroxaban, edoxaban, or a VKA-proxy medication) or two bottle openings (in case of apixaban or dabigatran-proxy medication) were registered, divided by the total number of monitored days and multiplied by 100. To correct the adherence estimates, bottle openings for refills or medication interruptions allowed by a physician were taken into account. To analyze the clinical effect of non-adherence between OD and BID, the number of unprotected days was calculated based on the simulation of *Vrijens *et al*.: i.e.,* ≥ 1 or ≥ 3 consecutive missed doses for OD or BID respectively, or any extra doses over the dosing regimen in a time interval of 24 h, were considered as an ‘unprotected day’ [11].

Patients with a regimen adherence < 80% were categorized as “low adherence”, following the definition most used in the literature [6]. The “low adherence” patients received additional adherence telemonitoring for three months immediately after their regular adherence monitoring period. With telemonitoring, the data of the MEMS are transferred immediately by wireless transmission to an Internet server (through an NFC-compatible smartphone). Accordingly, telephone feedback was given in case of intake irregularities (i.e., for OD medication, more than one intake over 24 h or one missed dose; for BID medication, more than two intakes over 24 h or three consecutive missed doses). We have shown before that such telemonitoring with rapid feedback improves adherence [11, 12].

A small sample of AF patients randomized to the SC group was also monitored using a MEMS cap. This sample was kept as small as possible not to trigger too many patients in the control group to focus on their adherence on top of usual care, which would constitute a bias-by-measurement (Hawthorne effect) for the trial [13]. In addition, the MEMS cap in this group was not equipped with an LCD screen. Based on a standard deviation of regimen adherence in a control group of ± 20% [12] and a confidence interval of 5%, a minimum of 64 SC patients should be monitored with the MEMS by the end of the trial. Our goal was to measure OAC therapy adherence in a real-world setting with as little interference bias as possible. These SC patients were randomly selected by consecutive inclusion when MEMS caps without LCD screens were available and stratified to OAC medication.

To further assess the effect of using the MEMS on therapy adherence in the SC group, 24 additional SC patients received MEMS recording without an LCD screen after a prior period of three months during which pill count was performed: taking adherence (i.e., the proportion of prescribed doses taken) was compared between pill count and MEMS measurements.

### Impact of COVID-19 on trial execution and on the methods described

The COVID-19 pandemic hit while the AF-EduCare trial and app sub-study were ongoing. During the period from March 2020 to March 2021, there were several periods with restrictions, such as lockdowns, where patients were only allowed to come to the hospital for urgent matters. Due to the restrictions, patients could not always comply with the study visits and used the medication bottle for longer periods than planned. An overview of the number of patients who had an M1 or M2 visit during this period is presented in Supplementary Table 1. To intervene with the proposed timeline of the patients as little as possible, outdoor drive-ins were organized. During these drive-ins, patients drove to the hospital parking lot; the MEMS was safely accepted and read out. After the reading, the patients were given a go if therapy adherence was higher than 80%, or the necessary equipment for adherence telemonitoring (MEMS with smartphone) was given if regimen adherence was < 80%.

### Statistical analysis

Data were analyzed using IBM SPSS version 28.0 (IBM Corporation, Armonk, USA). Variables were described as numbers and percentages, mean ± standard deviation (SD), or median and interquartile range (IQR) as appropriate. Normal distribution was assessed using the Shapiro–Wilk test. For continuous variables, differences between two or more groups were compared using the Mann–Whitney *U* test or the Kruskal–Wallis test (non-parametric). The chi-squared test was used for categorical variables when appropriate. Bonferroni correction was used to correct for multiple comparisons after which *p* values < 0.05 were considered statistically significant.

## Results

### Demographics

**Fig. 1:**
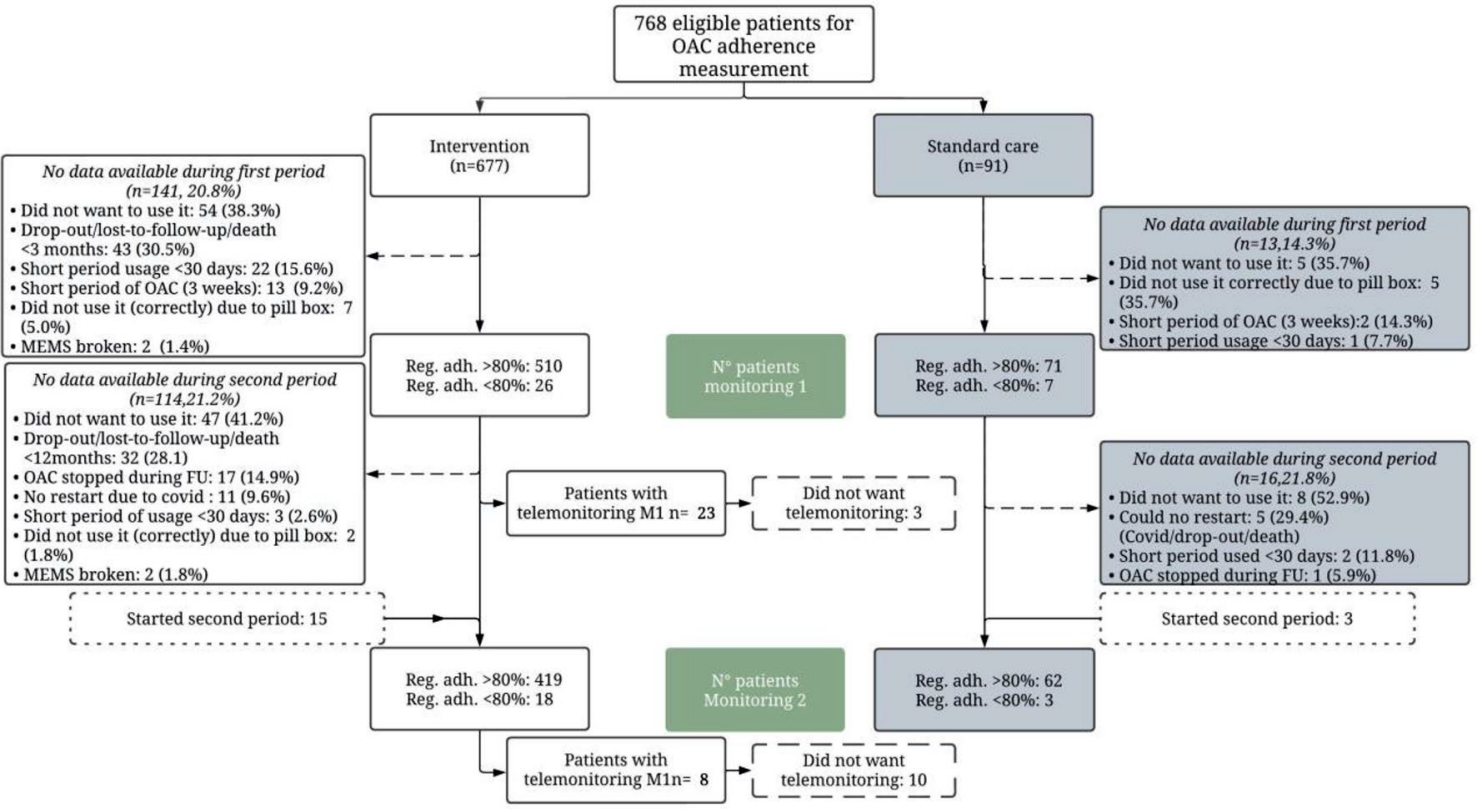
Flowchart of patients who were initiated with a MEMS for monitoring regimen adherence. Reg. adh: regimen adherence; MEMS: Medication Event Monitoring System, Monitoring 1 (M1): Baseline until 3 months; Monitoring 2 (M2): 12 to 15 months

A total of 768 patients of the AF-EduCare/AF-EduApp study were eligible for monitoring of therapy adherence (Fig. 1), of which 91 (11.8%) received standard care, and 677 (86.8%) received targeted education (i.e., 313 (40.8%) in-person, 224 (29.2%) online and 140 (18.2%) app-based education). No data of the MEMS was available for a total of 284 patients. The most frequent reason patients were excluded during both monitoring periods was refusal to use the medication bottle (114 of the 284 excluded patients; 40.1%) as patients claimed to continue their medication intake habits. Measurements of patients who used the medication bottle for less than 30 days for different reasons (e.g., technical issues, termination of medication…) were also excluded (10.6%).

The patients had a mean age of 70.1 ± 7.9 years, 69.7% were male, with a mean CHA_2_DS_2_-VASc score of 3.2 ± 1.6. A total of 72 (9.4%) patients were taking VKA, and 689 (89.7%) patients were on a NOAC. Another seven patients (0.9%) were not on any OAC at the start of the study but initiated OAC during the study, at which monitoring was also initiated (OAC initiated in first three months: 2 patients, OAC initiated after three months: 5 patients). The randomly selected sample of patients in the standard care group was well-matched with the intervention group (Table 1). In total, 614 patients were monitored successfully during M1, and 502 patients were monitored successfully during M2. A total of 18 patients only had a second period without a first measurement due to various reasons (i.e., no OAC at the start (*n* = 5), short period on OAC (*n* = 2), did not want to use MEMS (*n* = 7), < 30 days of use (*n* = 2), MEMS broken (*n* = 2)). Overall, 483 patients were followed during both monitoring periods.

**Table 1:** Baseline characteristics of the included AF patients

	Total population (*n* = 768)	Intervention groups (*n* = 677)	Standard care (*n* = 91)	*p* value*
Male, *n* (%)	535 (69.7)	472 (69.7)	63 (69.2)	0.924
Age (years), mean ± SD	70.1 ± 7.9	70.1 ± 8.0	70.2 ± 7.6	0.992
CHA_2_DS_2_-VASc score, mean ± SD	3.2 ± 1.6	3.2 ± 1.5	3.5 ± 1.6	0.227
HAS-BLED score, mean ± SD	1.5 ± 0.8	1.5 ± 0.8	1.6 ± 0.9	0.357
Time since AF diagnosis (years), mean ± SD	5.8 ± 7.0	5.8 ± 7.1	6.1 ± 6.1	0.501
Oral anticoagulation, *n* (%)				0.457
Apixaban	207 (27.0)	176 (26.0)	31 (34.1)	
Edoxaban	246 (32.0)	223 (32.9)	23 (25.3%)	
Rivaroxaban	167 (21.7)	148 (21.9)	19 (20.9)	
Dabigatran	69 (9.0)	61 (9.0)	8 (8.8)	
VKA	72 (9.4)	62 (9.2)	10 (11.0)	
OAC started during the study	7 (0.9)	7 (1.0)	0 (0.0)	

*AF* Atrial fibrillation, *SD* Standard DEVIATION, *CHA2DS2-VASc* Congestive heart failure (1), Hypertension (1), Age > 75 years (2), Diabetes mellitus (1), stroke (2), Vascular disease (1), Age 65–74 years (1), Sex category (female = 1); *HAS-BLED* Systolic blood pressure > 160 mmHg (1), Abnormal renal and/or hepatic function (1 point each), stroke (1), Bleeding history or predisposition (1), Labile INR (1), age > 65 years (1), Drugs or excessive alcohol drinking (1 point each); *VKA* Vitamin K Antagonist

^*^A Mann–Whitney *U* test was used for continuous data, and a chi-square test was used for categorical data

### Therapy adherence to OAC

**Fig. 2:**
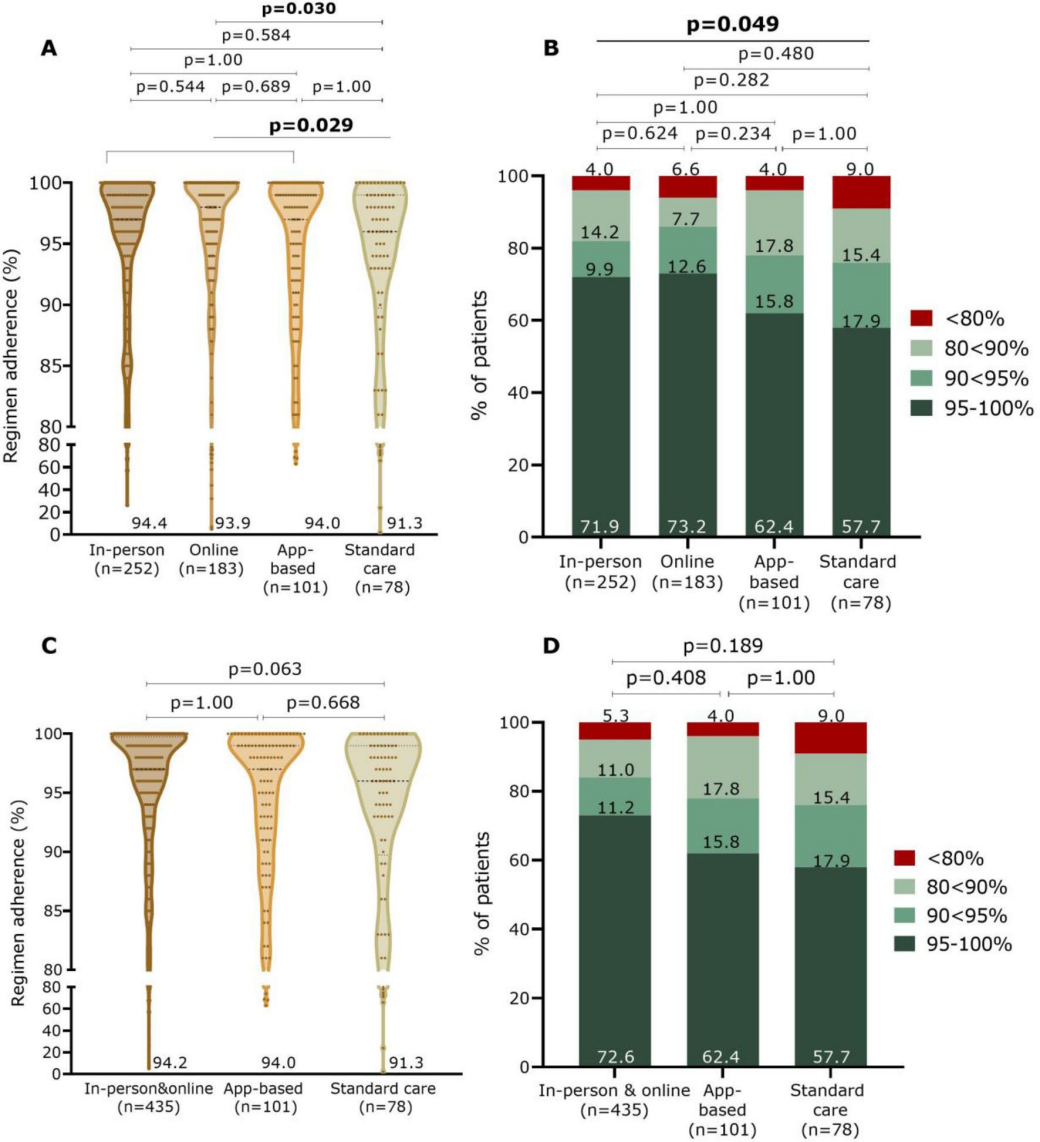
Regimen adherence to OAC between the study groups during the first monitoring period. Data are shown as violin plot with dots (**A**, **C**) and shown in four adherence categories (**B**, **D**)

During M1 (*n* = 614), overall regimen adherence was 93.8 ± 10.8% with a mean registration period of 100 ± 43.6 days. Patients in the intervention groups had a significantly higher regimen adherence than the SC group (94.2 ± 10.0% vs. 91.3 ± 15.0%; p = 0.029), driven by a significant higher therapy adherence of the Online group compared to SC (*p* = 0.030) (Fig. 2A). There was no significant difference between the different intervention groups (*p* = 0.148). Also, when looking at various adherence thresholds (i.e., 80%, 90%, and 95%), there was a significant difference between the four study groups (*p* = 0.049) (Fig. 2B). However, there were no significant differences when pairwise comparisons were performed between the four study groups. Visually, the app group seems to show an adherence more comparable to the SC group than to the other two intervention groups, but this could not be demonstrated statistically (Fig. 2C and D).

**Fig. 3:**
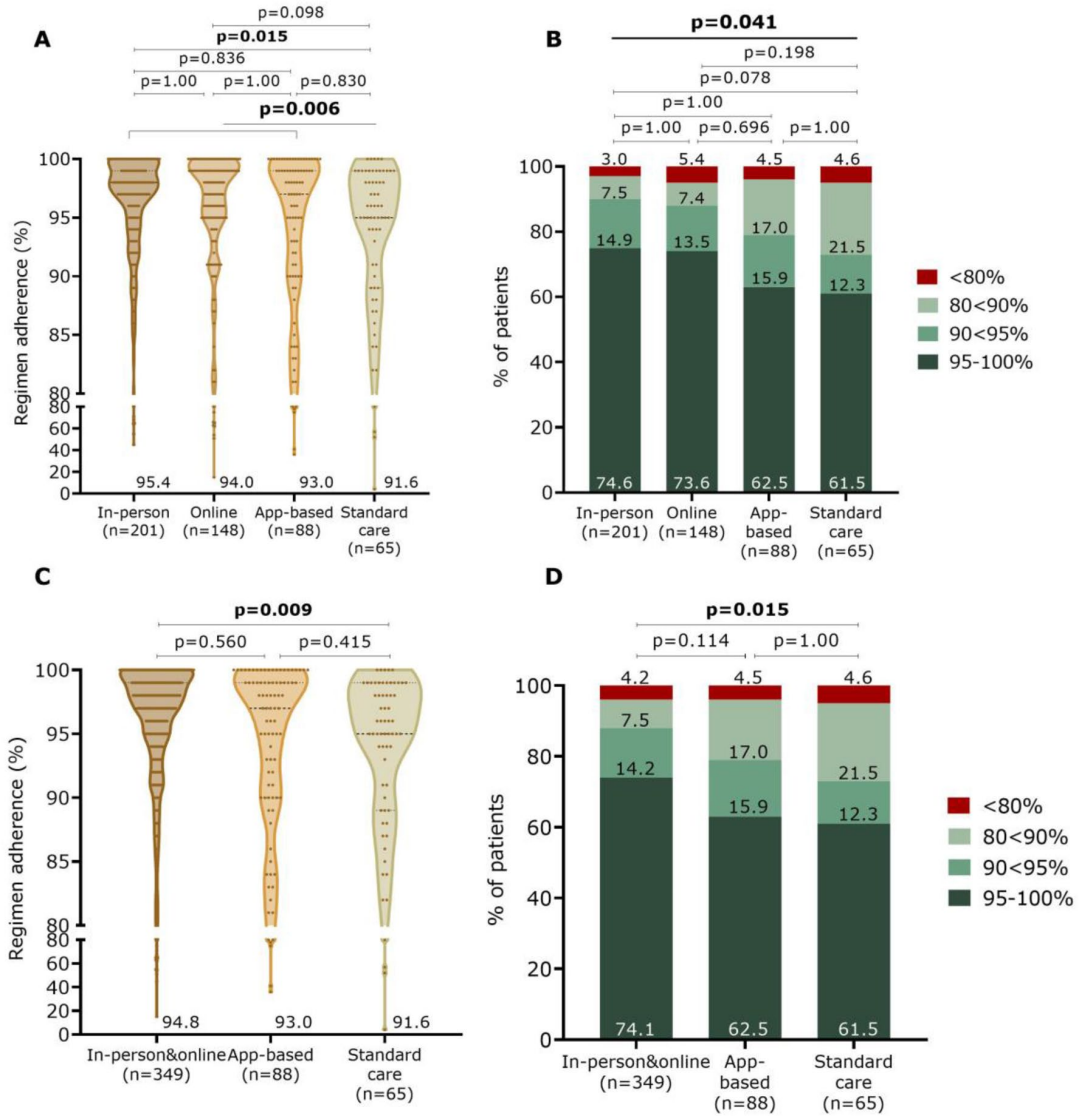
Regimen adherence to OAC between the study groups during the second monitoring period. Data are shown as violin plot with dots (**A** and **C**) and shown in four adherence categories (**B** and **D**)

Due to various circumstances, fewer patients had an M2 (Fig. 1). A total of 502 patients showed an overall regimen adherence of 94.1 ± 10.1% (mean registration duration of 107.2 ± 49.6 days). Again, patients in the intervention groups combined had significantly higher adherence than patients in the standard care group (94.4 ± 9.3% vs. 91.6 ± 14.0%; *p* = 0.006) (Fig. 3A). This was mainly driven by a significantly higher score in the in-person group (95.4 ± 7.3%) compared to the standard care group (*p* = 0.015). Therapy adherence of the app group did not significantly differ from both other study groups (93.0 ± 10.7 vs. 94.8 ± 8.9; *p* = 0.560) or the SC (91.6 ± 14.0; *p* = 0.415). Also, the combined in-person and online adherence was significantly higher than SC (*p* = 0.009) (Fig. 3C). Comparing categories of therapy adherence showed a significant difference between the four study groups (*p* = 0.041) (Fig. 3B), but again without significant differences with the pairwise comparison of the four study groups. Compared to SC, the combined online and in-person groups showed a significant difference (*p* = 0.015). The app group did not significantly differ from the other two intervention groups (*p* = 0.114) or the SC (*p* = 1.00) (Fig. 3D).

### Impact of telemonitoring

Of the 26 patients (out of 537 intervention patients, i.e., 4.8%) with a regimen adherence lower than 80% during M1, 24 patients completed a telemonitoring period of three months with daily follow-up and phone feedback by the study team if needed. This significantly increased their regimen adherence (from 60.0 ± 22.1% to 89.5 ± 12.3%; *p* < 0.001). Eighteen out of the 436 intervention patients (4.1%) had a regimen adherence lower than 80% during M2, of whom only eight patients completed a telemonitoring period, which significantly improved therapy adherence (from 51.4 ± 17.0% to 89.9 ± 9.7%; *p* = 0.012). The patients who did not start telemonitoring did not want to use the smartphone with NFC or did not want to use the MEMS again.

### Impact of AF-EduCare approach on therapy adherence to OAC over time

**Fig. 4:**
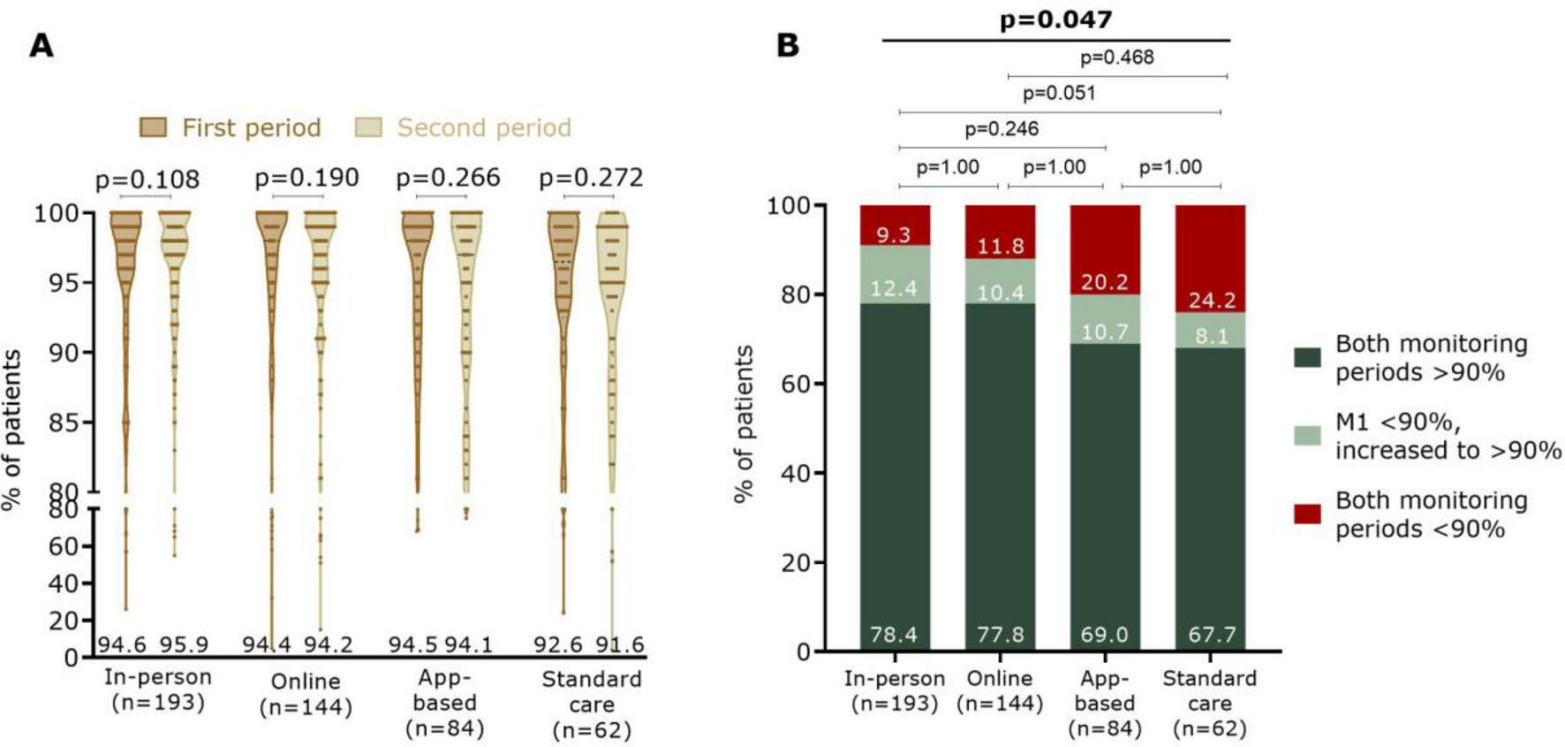
Regimen adherence to OAC over time between the study groups. Data are shown as violin plot with dots (**A**) and shown in four adherence categories (**B**)

A total of 483 patients fulfilled both monitoring periods. Overall, therapy adherence was preserved between the two monitoring periods (M1: 94.3 ± 9.8% vs. M2: 94.5 ± 9.1%; *p* = 0.538). In all groups separately, also in the SC, patients were able to sustain their therapy adherence over time (Fig. 4A). When making three categories of therapy adherence over time (i.e., patients who had a therapy adherence > 90% during both follow-up periods, patients with adherence < 90% during M1 and who improved above 90% at M2, or patients who did not improve > 90%), there were significant adherence differences between the study groups (*p* = 0.047) (Fig. 4B). Again, the app group seems to be more comparable with the SC and lower than the in-person and online combined. However, this could not be demonstrated statistically (*p* = 1.00 and *p* = 0.141, respectively). Only patients in the in-person and online combined scored better than patients in the SC group (*p* = 0.027). When looking at patients with therapy adherence < 90% during M1 (*n* = 120), Table 2 seems to suggest that education patients (especially in-person) improved more throughout the study than SC patients, although this did not reach statistical significance (*p* = 0.087).

**Table 2:** Optimal adherence after monitoring 1 (M1) and monitoring 2 (M2

	Total (*n* = 483)	In-person (*n* = 193)	Online (*n* = 144)	App (*n* = 84)	Standard care (*n* = 62)	*p* value*
≥ 90% adherence during both follow-ups	363 (75.2)	151 (78.4%)	112 (77.8)	58 (69.0)	42 (67.7)	0.165
< 90% at M1:	120 (24.8)	42 (21.8)	32 (22.2)	26 (31.0)	20 (32.3)	0.087
Same therapy adherence (± 5%)	31 (25.8)	10 (23.8)	6 (18.8)	9 (34.6)	6 (30.0)	
> 5% increased	47 (39.2)	23 (54.8)	12 (37.5)	7 (26.9)	5 (25.0)	
> 5% decreased	42 (35.0)	9 (21.4)	14 (43.8)	10 (38.5)	9 (45.0)	

^*^Chi-square test

Patients who were motivated to use the MEMS during two monitoring periods had a higher therapy adherence at M1 (94.3 ± 9.8%) compared to patients who only used it during M1 (92.0 ± 13.9; *p* = 0.060) (Table 3), with good preservation of this high adherence over time (M1: 94.3 ± 9.8% vs. 94.5 ± 9.1; *p* = 0.538; Table 3). When looking at the app group, only 37 of the 84 patients (44.0%) were motivated enough to use the MEMS during the whole study, as prescribed per protocol. These patients had a significantly higher mean therapy adherence (96.2 ± 5.3%) during M2 compared to the 47 patients who used the MEMS only from 12 to 15 months (92.5 ± 7.1%; *p* = 0.048). This difference was already emerging at M1, although not yet significant (96.3 ± 4.8% vs. 93.1 ± 8.0%; *p* = 0.067).

**Table 3:** Patient motivation to use the MEMS versus their therapy adherence

	All four groups			Application driven		
	One monitoring (*n* = 131)	Both monitorings (*n* = 483)	*p* value	Two separate monitorings* (*n* = 47)	Whole study period* (*n* = 37)	*p* value
Therapy adherence, mean ± SD	92.0 ± 13.9	94.3 ± 9.8	0.060	93.1 ± 8.0%	96.3 ± 4.8%	0.067
Therapy adherence, mean ± SD		94.5 ± 9.1		92.5 ± 7.1%	96.2 ± 5.3%	**0.048**
*p* value		0.538		0.675	0.717	

^*^Two separate monitorings: patients who used the medication bottle during the two monitoring periods separately vs. Whole study period: patients who used the medication bottle during the full 15 months of study period; *P* values were calculated with the Mann–Whitney *U* test with in bold *p* < 0.05

### High therapy adherence in standard care

A group of 22 SC patients was evaluated by both pill count and MEMS monitoring. Eight had to be excluded because they did not fulfill the requirements for either the MEMS or pill count data set, and four more had to be excluded as they had forgotten to bring all their remaining OAC medication to the pill count appointments. In the remaining 10, there was a significant difference in taking adherence as measured by pill count or MEMS usage, 92.3 ± 6.8% vs. 98.3 ± 3.4%, respectively; *p* = 0.019.

### Adherence of once vs. twice daily taken OAC and its potential therapeutic impact

During both monitoring periods, OD OAC regimen adherence was significantly higher compared to BID adherence (M1: OD 94.6 ± 9.9% vs. BID 92.3 ± 12.1%, *p* < 0.001; M2: OD 94.9 ± 9.8% vs. BID 92.5 ± 10.9%, *p* < 0.001). However, when looking at unprotected days based on the manuscript of *Vrijens *et al*.*[11], there was a trend of BID having significantly fewer unprotected days than OD (4.1 ± 9.0 vs. 5.5 ± 10.5 unprotected days, respectively, *p* = 0.050) at M1. This was confirmed during M2 with 3.1 ± 6.1 vs. 5.5 ± 10.2 unprotected days for BID and OD OAC regimens, respectively; *p* = 0.002.

## Discussion

This report is the first large prospective study investigating adherence for all OACs using electronic monitoring. The results show that AF patients generally had a very high regimen adherence (94.3 ± 9.8% and 94.5 ± 9.1% at first, respectively, second measurement period, one year apart). This could be related to the fact that all patients consented to participate in a clinical trial, presumably preselecting more eligible patients. Nevertheless, educational intervention optimized adherence, but often not statistically significant when delivered via an app. In addition, we confirm prior findings that MEMS combined with tele-monitoring and immediate phone-based feedback leads to a very significant improvement in those with an initial adherence of < 80%. Finally, while regimen adherence is higher in OD than in BID, the projected steadiness of clinical protection may not be different or even better for BID regimens.

### Therapy adherence to OAC

With the advent of NOACs, guidelines have stressed the importance of correct intake, i.e., regimen adherence. Different studies illustrate that this increased focus leads to improvements. A recently published study in a large cohort of more than 500.000 AF patients from European countries, newly started on NOAC, showed an increase in the proportion of those with a Medication Possession Ratio (MPR) ≥ 90% after one year from 62% in 2011 to 80% in 2017 [14]. Likewise, a study using prescription data of NOACs from Sweden (*n* = 5.254) and the Netherlands (*n* = 430) found that 86.6% and 87.2% of the patients, respectively, had an MPR > 90% [15]. A study in a Belgian cohort of 766 patients showed a median MPR of 95.2% (IQR = 87.8–99.7), although 31% had an MPR < 90% [16]. Another recently published large cohort study in Belgium (> 200.000 patients) based on prescription data showed that almost 90% of NOAC patients had a high proportion of days covered (PDC, > 90%), with a mean PDC after one year of 97.3% ± 5.8% in persistent users [17]. Nevertheless, MPR and PDC mostly overestimate therapy adherence compared to regimen adherence, as they do not consider the number of correct doses on each day. The very high adherence in our study, therefore, is reassuring. It is comparable to two other studies using electronic monitoring, which reported values of 91.9% and 93.8% using similar technology [12, 18]. It is clear that adherence to NOAC therapy has improved over the years in many countries. Apart from the study context, our high values may also reflect that Belgium is one of the leading countries in terms of guideline adherence for OAC treatment. This confirms studies in other therapeutic domains, in which Belgian patients showed a higher therapy adherence than other European countries [2, 19].

### Impact of telemonitoring

Regardless of the overall high regimen adherence, about 4–5% of the patients were clearly sub-adherent (< 80%). Telemonitoring with feedback significantly improved their regimen adherence during both follow-up periods, indicating that this technique is an option for low-adherence patients. In a prior study by our group in which telemonitoring-based telephone feedback was given to all patients, regimen adherence increased from 93.8 to 96.8% (*p* = 0.002) [12]. Despite telemonitoring, mean adherence in the patients with < 80% adherence in this trial did not improve to > 90% (only to 89.5 ± 12.3% and 89.9 ± 9.7% during periods 1 and 2, respectively), i.e., the generally considered threshold for effective therapy to prevent adverse outcomes (e.g., stroke, major bleeding, myocardial infarction) [20]. Nevertheless, 16 of 24 and 6 of 8 individual patients improved beyond this threshold.

### Impact of AF-EduCare approach on therapy adherence to OAC in general and over time

Patients in the intervention groups with targeted education scored significantly higher during both monitoring periods than the SC group. The higher adherence in the education groups might be related to the education itself and/or the use of MEMS with an LCD screen providing direct patient feedback. The high therapy adherence during the first monitoring period showed an early impact, which was maintained after one year when the second monitoring was performed.

Two other studies have evaluated the impact of education on adherence to NOAC therapy. In the AEGEAN study, 1228 AF patients, who were NOAC naïve and initiated on apixaban, were included at different European centers and randomized to SC or an education program (Edu) [18]. The education program consisted of an education booklet on AF and OAC treatment, access to reminder tools, and access to a virtual clinic that called the patients regularly. After 24 weeks, patients in the Edu group were re-randomized to further education or a secondary SC group for again 24 weeks. Adherence was measured using electronic monitoring as in our study, albeit with a different medication holder (Helping Hand, WestRock Switzerland Ltd., Sion, Switzerland). Mean regimen adherence in the Edu group was 91.9 ± 16.1% after 24 weeks and 90.4 ± 18.0% after 48 weeks, slightly lower than in our study. No significant difference (*p* > 0.07) was seen between the groups at 24 weeks (Edu: 91.9 ± 16.1%; SC: 91.6 ± 17.1%) or 48 weeks (Edu: 90.4 ± 18.0%; SC: 90.1 ± 18.6% secondary SC: 89.3 ± 18.1%). Another study investigated the effect of a pharmacist-led educational intervention on regimen adherence for NOAC [21]. A total of 301 patients were included in the SMAAP-AF study who received electronic monitoring (i.e., a card-type electronic device attached to the press-through package) to begin with an observational phase. Afterward, they were randomized to standard care and educational intervention. Despite a significant increase in therapy adherence in both groups, the mean change in medication adherence over time was not significantly different between the two groups (i.e., SC: 2.9% [7.5%] vs. education 3.4% [8.3%]). As in the AF-EduCare study, AEGEAN and SMAAP-AF patients in the SC group had very high regimen adherence (i.e., 91.6 ± 17.1% after 24 weeks and 90.1 ± 18.6% after 48 weeks in AEGEAN, and 94.5% and 92.9% in SMAAP-AF), making it challenging to demonstrate that education improves therapy adherence. Nevertheless, unlike AEGEAN and SMAAP-AF, our intervention groups scored significantly higher than the SC group during both monitoring periods. This may be related to the personalized and targeted education on AF in general and its management used in our study.

When comparing the different educational follow-up strategies, in-person education had the highest impact on therapy adherence, with 90.8% of patients having a therapy adherence > 90%. The app-driven educational follow-up strategy (i.e., education via an application and the possibility to install medication reminders in the application) scored lower compared to the other two education groups, with only 79.7% of patients with a therapy adherence > 90% (*p* = 0.114). The lower therapy adherence could be related to app fatigue during follow-up. This was indeed seen in the pilot study, with a significant decrease in app usage after one month [10]. Less app-driven education and feedback could lead to fewer patients taking their medication correctly compared to the in-person group who regularly saw the study nurse. Although the online group has a similar number of per-protocol in-person visits as the app group, the online group seemed to score better than the app group and similar to the in-person group. The findings may be confounded by the fact that more patients in the app group (67.3%) had their first monitoring period during COVID-19 peaks compared to the in-person (29.2%) and online (31.7%) groups. As a result, these patients had more telephone follow-ups instead of in-person visits, which could result in less adequate feedback and stimulation for good therapy adherence. Based on all the combined results, it seems important to have a minimum number of personal visits to successfully motivate patients to better adherence rates. It is also good to note that due to the educational follow-up, more than 10% of the patients improved their therapy adherence to > 90% after one-year follow-up.

A reason for the high regimen adherence in all trials could be using an electronic device, leading to an overestimation of adherence due to the awareness of being monitored (i.e., the Hawthorne effect [13]). This was confirmed by our data in a subset of SC patients which showed an overestimation of 6% when adherence was based on electronic monitoring vs. pill count. *El Alili *et al*.* (8%) also reported this in a recent review [22]. Other reasons for high therapy adherence could be related to an increased focus on integrated care and patient-centricity in general (i.e., the type of standard care in which the patient is a central figure and more involved in their care), selection bias of people that consented to participate in a clinical trial, or the high exclusion rate of 35.7% who did not want to use the MEMS.

### Adherence of once vs. twice daily taken OAC and its potential therapeutic impact

Regimen adherence to OACs with an OD regimen was significantly higher (albeit minor) than those with a BID regimen during each monitoring period, which was also shown by several other studies [12, 17, 23]. The difference is small and should not preclude other adherence-improving measures (like education) to improve adherence for both OD and BID OACs. Paradoxically, OD NOACs may result in lower clinical protection, as was implicated by *Vrijens *et al., who simulated that missing one OD dose is equal to missing three consecutive BID doses [11]. The study of *Desteghe *et al*.* already showed fewer unprotected days (based on this definition) in BID regimens compared to OD [12]. In our study, patients on BID had significantly fewer unprotected days compared to OD regimen. These results show the important difference between regimen adherence and the clinical relevance of unprotected days. Further studies on clinical outcomes are needed to investigate this effect further.

### Usability of MEMS

Despite the usability of the MEMS to monitor therapy adherence to OAC, various patients still do not want to use it due to multiple reasons. Of the 744 patients needing to be started with MEMS monitoring, only 484 (65.1%) completed both monitoring periods. Nevertheless, 615 patients (82.7%) used MEMS correctly for three months during the first monitoring period. Hence, the non-use during the second period may be more related to patient fatigue than inherent problems with the technology itself [24]. We have shown that patient motivation for MEMS relates to regimen adherence to OAC. Whether that is a causal or coincidental finding merits further study. In any case, MEMS proves to be a viable tool for monitoring and increasing patients' awareness of adherence and possible pitfalls.

### Limitations

The MEMS is a good tool for monitoring therapy adherence. Nevertheless, it requires extra effort from the patients who, for example, use a medication pill box or another medication intake system, given that the medication to be monitored has to be put in the bottle. The high drop-out rate due to the use of the MEMS could also bias the high adherence rate in this study. In addition, using this MEMS somehow alarmed the patient for being observed, which could improve therapy adherence compared to regular medication taking. This makes it more difficult to objectively measure adherence in the standard care group which could affect the comparison of the standard care group with the intervention groups. With this study, it was shown that telemonitoring led to a significant improvement. However, this was performed in only a small sample of patients who had therapy adherence < 80%. It remains hard to assess the impact of COVID-19 on our study. Despite all mitigation efforts, it certainly reduced personal contact with the patients through which it may have affected findings.

## Conclusions

Based on the results of this study, this Belgian AF population, in general, had already a very high regimen adherence to OAC intake. Nevertheless, an educational intervention further optimized adherence (albeit less so when delivered via an app with less in-person contact) and could prevent attrition of adherence. Monitoring therapy adherence is an additional educational tool to provide insights into the structural causes of non-adherence, while telemonitoring is particularly useful in AF patients with very low adherence (< 80%). The extent to which mHealth tools can replace in-person visits to maintain therapy adherence should be further investigated.

### Supplementary Information

Below is the link to the electronic supplementary material.

Supplementary file1 (DOCX 651 KB)

## Data Availability

The raw data supporting the conclusions of this article will be made available by the authors upon request.

## Abbreviations

AF: Atrial fibrillation
BID: Twice-daily taken
EHRA: European Heart Rhythm Association
ESC: European Society of Cardiology
M1: First monitoring period (baseline until 3 months)
M2: Second monitoring period (from 12 until 15 months)
MEMS: Medication Event Monitoring System
MPR: Medication Possession Ratio
NOAC: Non-vitamin K antagonist oral anticoagulant
OAC: Oral anticoagulation
OD: Once-daily taken
RF: Risk factor
SC: Standard care
VKA: Vitamin K antagonist
