# Supplementary material for: Adherence to oral anticoagulation measured by electronic monitoring in a Belgian atrial fibrillation population
Source: Clin Res Cardiol. 2023 Jul 27;112(12):1812–23. doi: 10.1007/s00392-023-02261-w (PMC10698080; doi:10.1007/s00392-023-02261-w)
Supplement: Supplementary file 1 — Supplementary file1 (DOCX 651 KB) [file 392_2023_2261_MOESM1_ESM.docx]

**Adherence to oral anticoagulation measured by electronic monitoring in a Belgian atrial fibrillation population**

Lieselotte Knaepen^abcd*1^, Michiel Delesie^abc*^, Johan Vijgen^cd^, Paul Dendale^cd^, Joris Ector^e^,
Lien Desteghe^abcd^, Hein Heidbuchel^abc^

a Antwerp University Hospital, Drie Eikenstraat 655, 2650 Edegem, Belgium

b Research Group Cardiovascular Diseases, University of Antwerp, Prinsstraat 13, 2000 Antwerp, Belgium

c Faculty of Medicine and Life Sciences, Hasselt University, Martelarenlaan 42, 3500 Hasselt, Belgium

d Heart Center Hasselt, Jessa Hospital, Stadsomvaart 11, 3500 Hasselt, Belgium

e: Department of Cardiology, University Hospitals Leuven, Leuven

## Supplementary material

### Supplementary Figure 1

S. Figure 1: Short study design AF-EduCare/AF-EduApp study


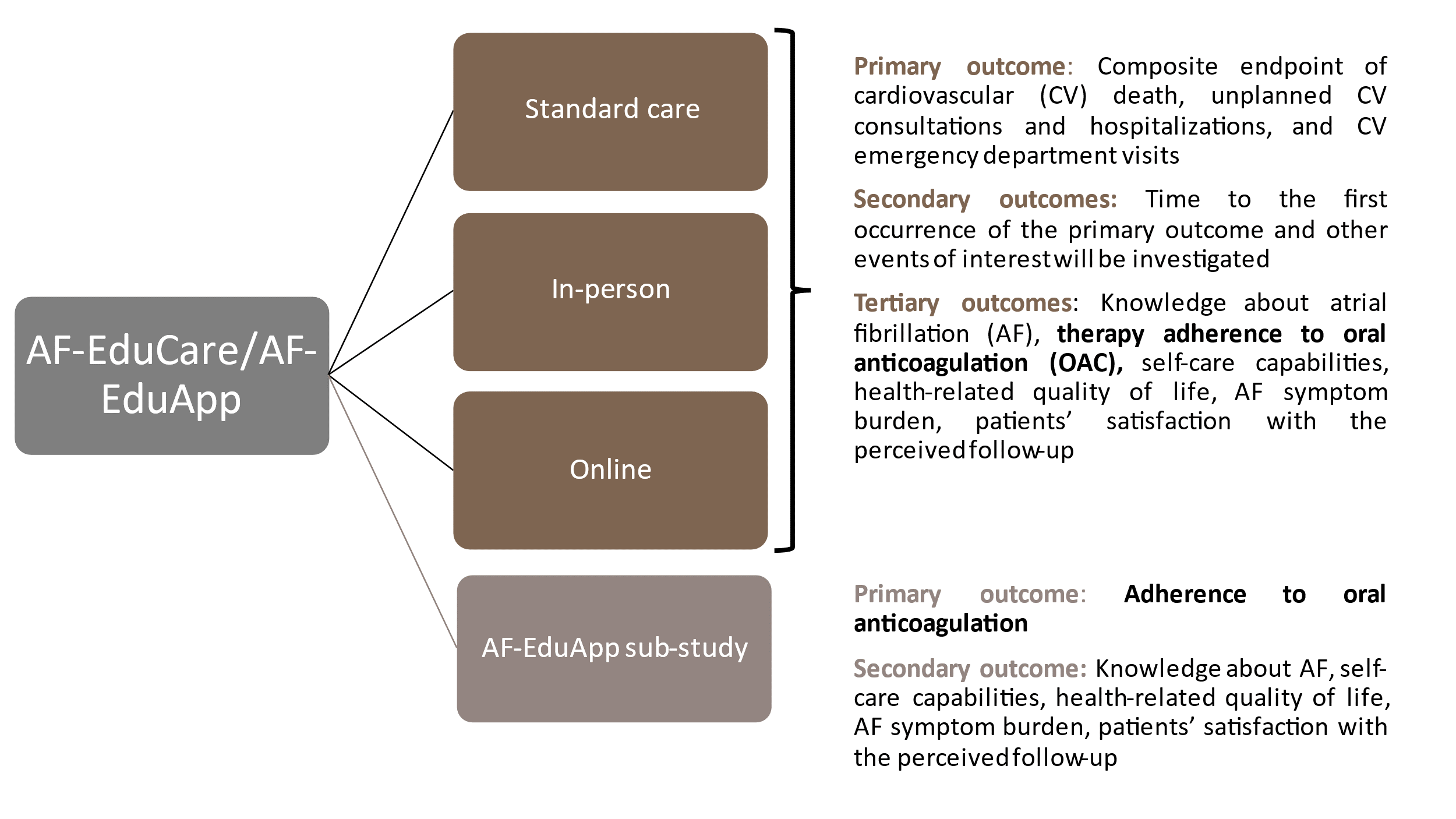


### Supplementary annex 1

The AF-EduApp is an in-house developed application validated by experts in the field and AF patients. The educational application is available for smartphones and tablets on IOS or Android. The app contains six main modules (S. Figure 2): i.e. an **Education** module with information, available 24/7, about AF and its treatment, a **Questionnaire** module, including questionnaires to assess patients’ knowledge about AF and AF-related risk factors; the a **Medication** module in which patients could add their medication list and have the possibility to activate interactive reminders, a **Measurement** module to keep track of parameters such as heart rate, blood pressure or weight, an **Appointment** module to include scheduled appointments, and a **Question** module via which patients could ask questions directly to the study nurse and a phone number of the study team is available for urgent questions. The patients could use the application according to their needs throughout the study, except when questionnaires were available.

S. Figure 2: Main screen AF-EduApp


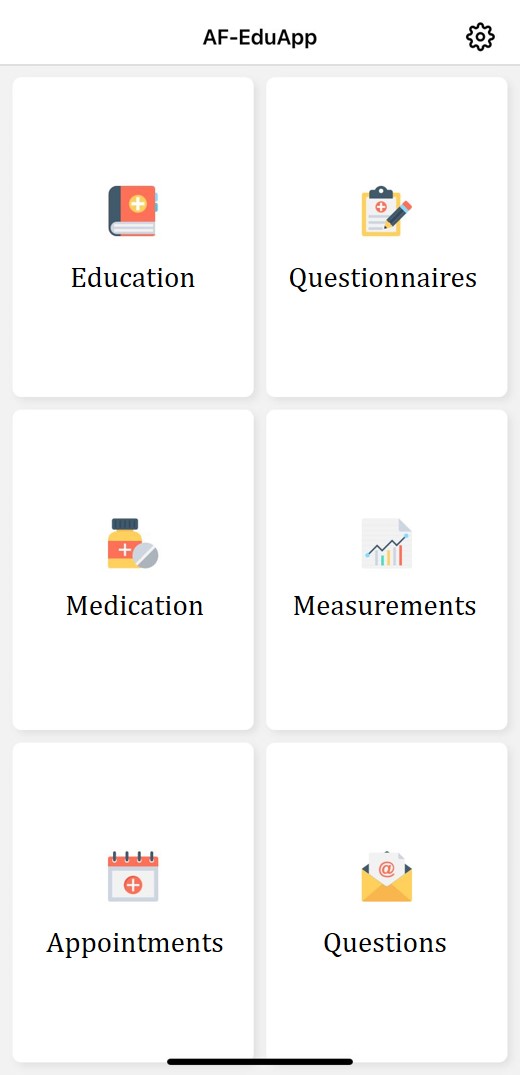


### S. Table 1

| **Table 1:** Number of patients who had their M1 or M2 visit during the COVID-19 pandemic (03/2020 – 03/2021) | | | | |
| --- | --- | --- | --- | --- |
| **M1 during COVID-19; n(%)** | **In-person (n=253)** | **Online (n=183)** | **App-based (n=101)** | **Standard care (n=78)** |
|  | 74 (29.2) | 58 (31.7) | 68 (67.3) | 17 (21.8) |
| **M2 during COVID-19; n(%)** | **In-person (n=201)** | **Online (n=148)** | **App-based (n=88)** | **Standard care (n=65)** |
|  | 131 (65.2) | 97 (65.5) | 17 (19.3) | 46 (70.8) |
| M1: monitoring 1 visit; M2: monitoring 2 visit | | | | |
